# Evaluation of Tissue-C and CorneaMax organ culture media for microbial contamination detection in corneal grafts using the BD BACTEC FX system

**DOI:** 10.1007/s10096-026-05459-8

**Published:** 2026-04-27

**Authors:** Judith Samard, Martin Fayolle, Sandrine Ninotta, Sophie Acquart, Chloe Talon, Manon Lleres-Vadeboin, Philippe Gain, Gilles Thuret, Paul O. Verhoeven, Anne Carricajo

**Affiliations:** 1 Department of Infectious Agents and Hygiene, Bacteriology-Hygiene Unit, University Hospital of St-Etienne, St-Etienne, France; 2 Faculty of Medicine, Jean Monnet University, St-Etienne, France; 3 CIRI, Centre International de Recherche en Infectiologie, GIMAP team, INSERM U1111 - CNRS UMR5308 - ENS Lyon - UCBL1, Lyon, France; 4 Eye Bank, Auvergne Rhône Alpes French Blood Center, Saint-Etienne, France; 5 Laboratory Biology, Engineering and Imaging for Ophthalmology, Faculty of Medicine, University Jean Monnet, Saint-Etienne, France; 6 Ophthalmology Department, University Hospital of St-Etienne, Saint-Etienne, France

**Keywords:** Corneal grafts, Organ culture medium, Tissue-C, CorneaMax, Alchimia, Eurobio, Microbiological testing, Becton Dickinson, BACTEC FX, Blood culture

## Abstract

**Purpose:**

Corneal grafting requires reliable organ culture conditions and robust microbiological monitoring. CorneaMax (Eurobio) and Tissue-C (Alchimia) are widely used in European eye banks, but the CE certification of CorneaMax was recently discontinued, prompting a transition to alternative media. This study assesses the performance of the BD BACTEC FX system in detecting microbial contamination in Tissue-C compared with CorneaMax, following European Pharmacopoeia standards.

**Methods:**

The organ culture media were inoculated with ten microorganisms in compliance with the European Pharmacopoeia, 10.5th edition. Different volumes (10, 1 or 0,2 mL) were inoculated into four BD BACTEC blood culture bottles, including Plus Aerobic/F, Lytic Anaerobic/F, Mycosis IC/F, and Plus Anaerobic/F (Becton Dickinson).

**Results:**

Using a 10-mL volume the sensitivity of microorganism detection across the four bottle types was 93.3% for CorneaMax and 66.7% for Tissue-C. Reducing the volume to 1 mL increased the sensitivity to 100% for CorneaMax and 80% for Tissue-C, although *S. pyogenes* and *C. acnes* remained undetected in Tissue-C. Further decreasing the volume to 200 µL ensured consistent detection of both species across three replicates. Interestingly, Tissue-C exhibited higher antibiotic activity than CorneaMax, likely explaining the reduced sensitivity of the method with Tissue-C compared to CorneaMax. Moreover, resin-free Lytic Anaerobic/F bottle provided no added value at any inoculation volume.

**Conclusion:**

This study demonstrates that microbiological monitoring of corneal grafts must be tailored to the culture medium. CorneaMax provides reliable detection with three BD BACTEC bottles, whereas Tissue-C, due to its higher antibiotic activity, requires further optimization of the monitoring strategy.

**Supplementary Information:**

The online version contains supplementary material available at 10.1007/s10096-026-05459-8.

## Introduction

Corneal grafting is one of the most commonly procedures tissue grafting performed worldwide, with approximately 185,000 transplants conducted annually [1, 2]. Despite a high success rate, one of the major complications of corneal grafting is post-keratoplasty infectious keratitis (PKIK). The incidence of PKIK in developed countries ranges from 0.02% to 7.9% [3–7] and, in some cases may progress to endophthalmitis [4, 8, 9]. Globally, Gram-positive bacteria are the most frequently identified pathogens, accounting for up to 83.3% of cases [5, 6, 10]. Within this group, *Staphylococcus aureus*, *Streptococcus pneumoniae* and coagulase-negative staphylococci such as *S. epidermidis*, are the predominant species and commonly found in the skin or oral microbiota. Gram-negative pathogens, led by *Pseudomonas aeruginosa*, are implicated in 10% to 25% of cases [11]. In addition, the incidence of fungal infections has increased from 9.8% to 66.7% when comparing PKIK cases between 1989 and 1994 and 2009–2014 [11]. The *Candida* genus is responsible for 5% to 10% of cases [3, 12], while *Fusarium* spp. and *Aspergillus* spp. are identified less frequently [13, 14]. The epidemiology of causative pathogens likely varies based on transplantation aseptic conditions, geographic region, and patient immune status [14]. Therefore, eye banks play a crucial role in ensuring the microbiological safety of corneal grafts.

In France, the standard preservation method for corneal grafts is organ culture at 31 °C [15], which maintains endothelial integrity and allows storage for up to five weeks [16]. Organ culture media contain antimicrobial agents to minimize the risk of microbial contamination in donor corneas. Before corneal release, microbiological testing for pathogenic microorganisms must be performed either by the bank or by a bacteriology laboratory. Inoculating organ culture medium into blood culture bottles has proven effective for detecting pathogenic microorganisms [17–19], particularly when using the BD BACTEC FX blood culture system [20]. Tissue-C (Alchimia) and CorneaMax (Eurobio) are two widely used organ culture media in European eye banks. Recently, the manufacturer discontinued the CE certification of CorneaMax, thereby requiring eye banks to transition to an alternative organ culture medium.

Our study aimed to evaluate the detection performance of the BD BACTEC FX blood culture system inoculated with the Tissue-C organ culture medium compared to the CorneaMax medium, following the guideline of the European Pharmacopoeia.

## Materials and methods

### Bacterial strains and organ culture medium

Ten microorganisms were selected following the recommendations of the European Pharmacopoeia (supplement 10.5, Sect. 2.6.27). These included Gram-positive bacteria (*Staphylococcus aureus* ATCC 6538, *Bacillus subtilis* CIP 5262, *Kocuria rhizophila* ATCC 9341, *Streptococcus pyogenes* CTCB 1031, *Cutibacterium acnes *clinical isolate, *Clostridium sporogenes* CIP 7939), Gram-negative bacteria (*Pseudomonas aeruginosa* ATCC 9027, *Bacteroides fragilis* ATCC 25285), and fungi (*Candida albicans* ATCC 10237, *Aspergillus brasiliensis* ATCC 1727) [21]. Bacterial strains were stored at -80 °C in cryotubes (CRYO80/M, Mast diagnostic, Amiens, France). Strains were subcultured on blood agar (43049, Biomérieux, Marcy l’Etoile), or Sabouraud-chloramphenicol agar (43596, Biomérieux), as appropriate. Identification was confirmed by MALDI-TOF mass spectrometry (MALDI Biotyper Sirus, Bruker, Wissembourg, France).

**Fig. 1:**
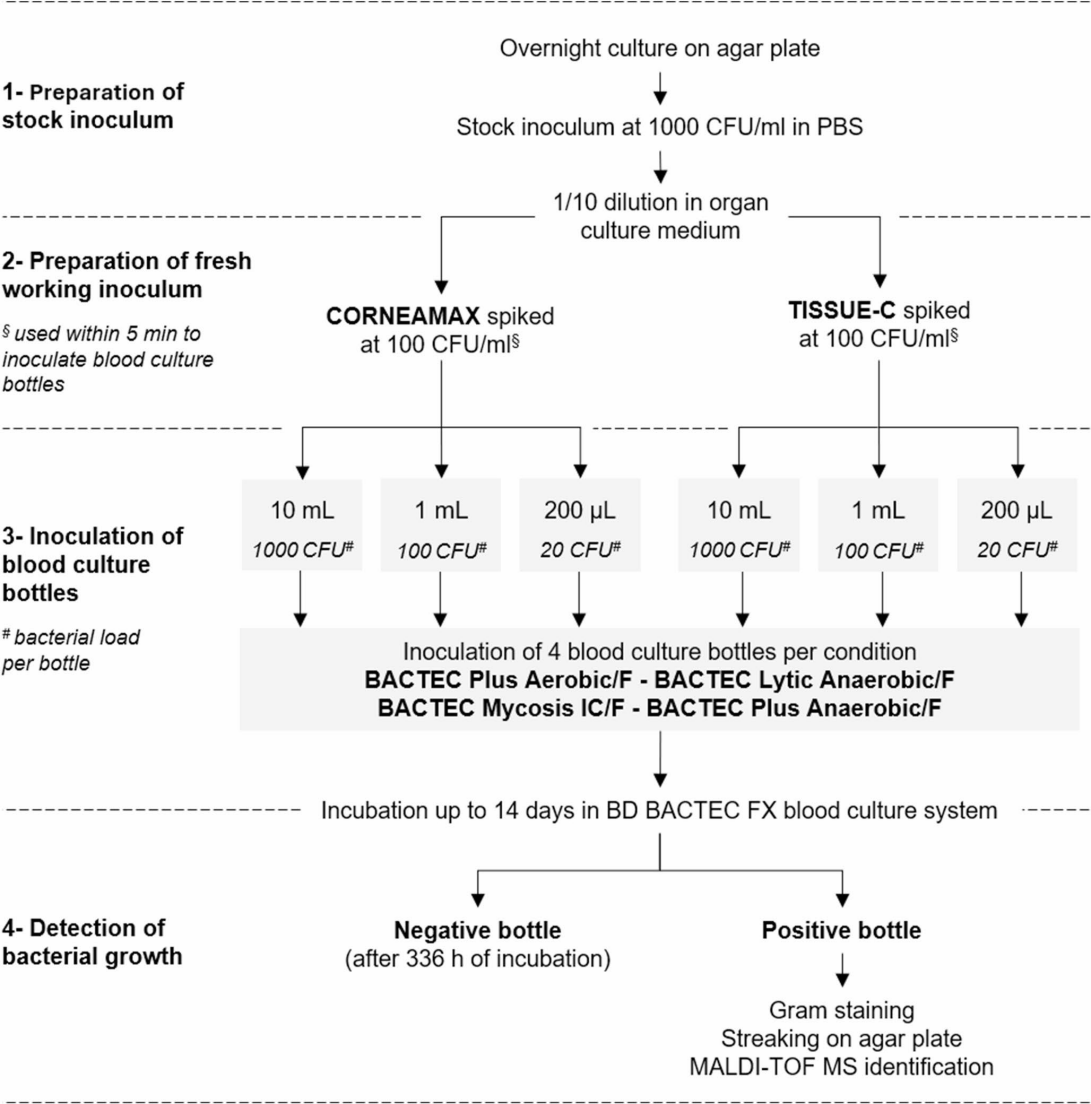
Experimental design of the study

For each strain, a 0.5 McF suspension was prepared in phosphate-buffered saline corresponding to approximately 10^8^ CFU/mL. This suspension was serially diluted to obtain a stock inoculum of 1000 CFU/mL (Fig. 1). A further tenfold dilution of the stock inoculum was performed in CorneaMax (Eurobio, Les Ulis, France) or Tissue-C (Alchimia, Ponte San Nicolò, Italy) organ culture media to obtain a working inoculum of 100 CFU/mL. The bacterial load of the stock inoculum was determined by using an automated plater (EasySpiral dilute, Interscience, Saint-Nom-la-Bretèche, France) and a colony counter (Scan 4000, Interscience).

### Microbiological testing procedure using blood culture bottles

A volume of 10 mL, 1 mL, or 200 µL of the working inoculum was inoculated into blood culture bottles including BACTEC Plus Aerobic/F, BACTEC Lytic Anaerobic/F, BACTEC Mycosis IC/F, and BACTEC Plus Anaerobic/F (Becton Dickinson and Company, Pont-de-Claix, France) (Fig. 1). The bottles were incubated in a BD BACTEC FX blood culture system (Becton Dickinson and Company) at 35 °C ± 1 °C for up to 14 days. The remaining organ culture media was incubated at 32 °C ± 1 °C (i.e. the temperature used to preserve cornea in eye bank) for 18 days to monitor any potential color change in the phenol red, indicating microbial growth [22, 23]. Positive bottles were plated on blood agar and Sabouraud-chloramphenicol agar and incubated overnight. Microbiological identification was performed by MALDI-TOF mass spectrometry (MALDI Biotyper Sirus, Bruker).

### Measure Antibiotic Activity of organ culture media

Antibiotic activity of organ culture media was assessed using microbiological plate diffusion assay as previously described with modifications [24, 25]. Briefly, we used three bacterial strains including *S. pyogenes* CTCB 1031 and *S. aureus* ATCC 6538, both susceptible to penicillin G and streptomycin, and *Enterococcus faecium* resistant to both antibiotics (see supporting information for details). Mueller-Hinton agar supplemented with defibrinated horse blood and β-NAD (MH-F) was uniformly inoculated with a tenfold dilution of 0.5 McF suspension. A well was punched into the agar and filled with 50 µL of either Tissue-C or CorneaMax. The antibiotic activity of the organ culture media resulted in an increased of the diameter of the inhibition zone around the well. The assay was repeated daily over five days, using the same media stored at room temperature and exposed to light, to assess potential degradation of antibiotic compounds. To confirm the reproducibility of the assay, the experiment was repeated on three different batches of media.

### Data analysis and statistics

All experiments were independently performed in triplicate on different days. The detection method was considered positive if at least one of the four bottles tested positive using the BACTEC System. Overall detection sensitivity was calculated for each culture medium and presented as a percentage. Statistical analysis was performed using MedCalc Statistical Software version 23.2.6 (MedCalc Software Ltd, Ostend, Belgium). Chi-squared test was used to compare detection sensitivity. Detection times for microorganisms were compared using a Wilcoxon signed ranks test. A p-value < 0.05 was considered statistically significant.

## Results

### Detection of microbial growth with a 10 mL volume of organ culture media

Since our routine microbiological procedure used 10 mL of organ culture medium to inoculate the bottles, we started with this volume to compare CorneaMax and Tissue-C media. Using 10 mL of fresh inoculum at a target concentration of 100 CFU/mL, each bottle was inoculated with approximately 1000 CFU (Fig. 1). For each of the three replicates, the bacterial load of the stock inoculum was quantified upon thawing (Table S1). Table 1 presents the detection sensitivity of each microorganism using an inoculation volume of 10 mL. With CorneaMax medium, all the anaerobic bacterial species and all the fungi tested were detected with at least one bottle. Among strict aerobic bacteria, *K. rhizophila* and *B. subtilis* were detected in only two out of three replicates. With Tissue-C medium, *K. rhizophila* and *B. subtilis* were also detected in only one replicate. Strikingly, *S. pyogenes* and *C. acnes* were not detected with Tissue-C. Considering all species tested, the sensitivity of the blood cultures for detecting microbes in 10 mL of organ culture media was respectively 93.3% (28/30), and 66.7% (20/30) for CorneaMax and Tissue-C media (*p* < 0.05, Chi-squared test).

**Table 1:** Detection of 10 microorganisms using blood cultures bottles inoculated with a 10 mL volume of organ culture medium artificially spiked at 100 CFU/mL

Microorganism species	Positive blood culture bottles inoculated with 10 mL of CorneaMax (% (*n*))						Positive blood culture bottles inoculated with 10 mL of Tissue-C (% (*n*))				
	Plus Aerobic/F		Lytic Anaerobic/F	Mycosis IC/F	Plus Anaerobic/F	Method sensitivity*	Plus Aerobic	Lytic Anaerobic/F	Mycosis IC/F	Plus Anaerobic/F	Method sensitivity*
Strict aerobic bacteria											
*K. rhizophila (n = 3)*		66.7 (2)	0 (0)	0 (0)	0 (0)	**66.7 (2)**	33.3 (1)	0 (0)	0 (0)	0 (0)	**33.3 (1)**
*P. aeruginosa (n = 3)*		100 (3)	0 (0)	0 (0)	0 (0)	**100 (3)**	100 (3)	0 (0)	0 (0)	0 (0)	**100 (3)**
Facultative aerobic-anaerobic bacteria											
*S. aureus (n = 3)*		100 (3)	0 (0)	0 (0)	100 (3)	**100 (3)**	33.3 (1)	0 (0)	0 (0)	100 (3)	**100 (3)**
*S. pyogenes (n = 3)*		100 (3)	0 (0)	0 (0)	100 (3)	**100 (3)**	0 (0)	0 (0)	0 (0)	0 (0)	**0 (0)**
*B. subtilis (n = 3)*		33.3 (1)	0 (0)	0 (0)	33.3 (1)	**66.7 (2)**	33.3 (1)	0 (0)	0 (0)	0 (0)	**33.3 (1)**
Strict anaerobic bacteria											
*B. fragilis (n = 3)*		0 (0)	100 (3)	0 (0)	100 (3)	**100 (3)**	0 (0)	100 (3)	0 (0)	100 (3)	**100 (3)**
*C. sporogenes (n = 3)*		0 (0)	0 (0)	0 (0)	100 (3)	**100 (3)**	0 (0)	33.3 (1)	0 (0)	100 (3)	**100 (3)**
*C. acnes (n = 3)*		0 (0)	0 (0)	0 (0)	100 (3)	**100 (3)**	0 (0)	0 (0)	0 (0)	0 (0)	**0 (0)**
Fungi											
*C. albicans (n = 3)*		100 (3)	0 (0)	100 (3)	0 (0)	**100 (3)**	100 (3)	0 (0)	66.7 (2)	0 (0)	**100 (3)**
*A. brasiliensis (n = 3)*		100 (3)	0 (0)	100 (3)	0 (0)	**100 (3)**	100 (3)	0 (0)	100 (3)	0 (0)	**100 (3)**
All microorganisms		60 (18/30)	10 (3/30)	20 (6/30)	53.3 (16/30)	**93.3** **(28/30)**	40 (12/30)	13.3 (4/30)	16.7 (5/30)	30 (9/30)	**66.7** **(20/30)**

*method sensitivity: detection of the pathogen was considered positive if at least one bottle was positive

By analyzing the results by bottle type, we found that the Lytic Anaerobic/F bottle had low sensitivity, detecting only 10% (3/30) of microorganisms in CorneaMax and 13.3% (4/30) in Tissue-C. In contrast, the Plus Anaerobic/F bottle, which contains an antibiotic-chelating resin, showed a sensitivity of 53.3% (16/30) in CorneaMax and 30% (9/30) in Tissue-C. Notably, the Plus Anaerobic/F bottle supported the detection of all strict anaerobes excepted *C. acnes* in Tissue-C medium. Interestingly, it also contributed to the detection of facultative anaerobes, including *S. pyogenes* in CorneaMax and *S. aureus* in Tissue-C. In addition, The Plus Aerobic/F bottle showed an overall sensitivity of 56.7% (17/30) in CorneaMax medium and 40% (12/30) in Tissue-C medium. Notably, it contributed to the detection of fungi, particularly in the Tissue-C medium, where the Mycosis bottle failed to detect *C. albicans* in one replicate.

### Detection of microbial growth with a 1 mL and 200 µL volume of organ culture media

Given that organ culture media contain very high concentrations of antibiotics, we hypothesized that the lack of sensitivity observed with 10 mL of Tissue-C was due to saturation of the chelation capacity of the resin-containing bottles. This hypothesis was also supported by the low sensitivity observed with the resin-free Lytic Anaerobic/F bottle. First, we tested a reduced inoculation volume of 1 mL of organ culture to improve antibiotic chelation in the resin-containing bottles (Table 2). Using 1 mL, each bottle was inoculated with approximately 100 CFU (Fig. 1). For each of the three replicates, the bacterial load of the stock inoculum was quantified upon thawing (Table S1).

**Table 2:** Detection of 10 microorganisms using blood cultures bottles inoculated with a 1 mL volume of organ culture medium artificially spiked at 100 CFU/mL

Microorganism species	Positive blood culture bottles inoculated with 1 mL of CorneaMax (% (*n*))					Positive blood culture bottles inoculated with 1 mL of Tissue-C (% (*n*))				
	Plus Aerobic/F	Lytic Anaerobic/F	Mycosis IC/F	Plus Anaerobic/F	Method sensitivity*	Plus Aerobic/F	Lytic Anaerobic/F	Mycosis IC/F	Plus Anaerobic/F	Method sensitivity*
Strict aerobic bacteria										
*K. rhizophila (n = 3)*	100 (3)	0 (0)	0 (0)	0 (0)	**100 (3)**	100 (3)	0 (0)	0 (0)	0 (0)	**100 (3)**
*P. aeruginosa (n = 3)*	100 (3)	0 (0)	0 (0)	0 (0)	**100 (3)**	100 (3)	0 (0)	0 (0)	0 (0)	**100 (3)**
Facultative aerobic-anaerobic bacteria										
*S. aureus (n = 3)*	100 (3)	33.3 (1)	0 (0)	100 (3)	**100 (3)**	66.7 (2)	33.3 (1)	0 (0)	100 (3)	**100 (3)**
*S. pyogenes (n = 3)*	100 (3)	0 (0)	0 (0)	100 (3)	**100 (3)**	0 (0)	0 (0)	0 (0)	0 (0)	**0 (0)**
*B. subtilis (n = 3)*	66.7 (2)	0 (0)	0 (0)	100 (3)	**100 (3)**	33.3 (1)	0 (0)	0 (0)	100 (3)	**100 (3)**
Strict anaerobic bacteria										
*B. fragilis (n = 3)*	0 (0)	100 (3)	0 (0)	100 (3)	**100 (3)**	0 (0)	100 (3)	0 (0)	100 (3)	**100 (3)**
*C. sporogenes (n = 3)*	0 (0)	66.7 (2)	0 (0)	100 (3)	**100 (3)**	0 (0)	66.7 (2)	0 (0)	100 (3)	**100 (3)**
*C. acnes (n = 3)*	0 (0)	0 (0)	0 (0)	100 (3)	**100 (3)**	0 (0)	0 (0)	0 (0)	0 (0)	**0 (0)**
Fungi										
*C. albicans (n = 3)*	100 (3)	0 (0)	100 (3)	0 (0)	**100 (3)**	100 (3)	0 (0)	66.7 (2)	0 (0)	**100 (3)**
*A. brasiliensis (n = 3)*	100 (3)	0 (0)	100 (3)	0 (0)	**100 (3)**	100 (3)	0 (0)	100 (3)	0 (0)	**100 (3)**
All microorganisms	66.7 (20/30)	20 (6/30)	20 (6/30)	60 (18/30)	**100** **(30/30)**	50 (15/30)	20 (6/30)	16.7 (5/30)	40 (12/30)	**80** **(24/30)**

*method sensitivity: detection of the pathogen was considered positive if at least one bottle was positive

Strikingly, *C. acnes* and *S. pyogenes*, which have low minimum inhibitory concentration (MIC) to penicillin G and streptomycin (Table S2), completely escaped detection in Tissue-C, thereby lowering the sensitivity of the method with this medium. Even at 10³ and 10⁴ CFU/mL, both species remained undetectable (data not shown), indicating that the failure arose from the medium itself rather than the inoculum. In addition, analysis by bottle type showed an increase in sensitivity with almost all bottles compared to an inoculation volume of 10 mL. In particular, resin-containing bottles, Plus Aerobic/F and Plus Anaerobic/F, were found to enhance the sensitivity of the method with both media, supporting the idea that residual antibiotic activity may impair the detection of the most susceptible microorganisms.

To further test this hypothesis, 200 µL of Tissue-C organ culture medium was inoculated into Plus Aerobic/F and Plus Anaerobic/F bottles to assess the detection of *C. acnes* and *S. pyogenes* (see Table S1 for quantification of the stock inoculum). Reducing the inoculation volume to 200 µL allowed the Plus Anaerobic/F bottle to consistently detect both species in both organ culture media, likely by minimizing residual antibiotic activity in the culture bottles.

### Antibiotic activity of organ culture media

Given the differences in sensitivity of the method with the two organ culture media, we next adapted a microbiological antibiotic assay to evaluate the antibiotic activity of CorneaMax and Tissue-C. Table 3 presents the results of the inhibition diameters obtained with the two organ culture media. As excepted, no inhibition zones were observed with the antibiotic-resistant *E. faecium* strain used as control. For *S. pyogenes* strain, the inhibition diameter reached 43 mm with Tissue-C and 28 mm with CorneaMax after 24 h of incubation, and similar results were observed with the *S. aureus* strain. Together, these results demonstrated that Tissue-C exhibit higher antibiotic activity than CorneaMax. This experiment was repeated using organ culture media stored at room temperature and within ambient light. Over the five-day testing period, inhibition zones decreased for both media, indicating a progressive loss of antibiotic activity.

**Table 3:** Evolution of antimicrobial activity of CorneaMax and Tissue-C media according to the storage duration at room temperature

Bacterial strain	Medium	Inhibition diameter (mm, or as a % if specified)					
		Storage duration at room temperature					Evolution from24 to 120 h (%)
		24 h	48 h	72 h	96 h	120 h	
*S. pyogenes* CTCB 1031	CorneaMax	28	27	26	26	18	-35.7
	Tissue-C	43	40	40	39	33	-23.6
*S. aureus* ATCC 6538	CorneaMax	28	28	28	27	25	-10.7
	Tissue-C	41	41	41	41	38	-7.3
E. faecium*	CorneaMax	6	6	6	6	6	-
	Tissue-C	6	6	6	6	6	-

*clinical strain resistant to streptomycin (MIC > 1024 mg/L) and to penicillin G (MIC = 32 mg/L)

### Time-to-detection of microbial growth

Finally, we investigated the impact of inoculum volume and organ culture medium on the detection time of microorganisms using the BD BACTEC FX system. The mean time-to-detection (mTTD) for the 10 microorganisms is summarized in Table 4. To compare the impact of inoculation volume, bottles that remained negative after 14 days of incubation were assigned a detection time of 336 h.

**Table 4:** Mean time-to-detection (mTTD) by BD BACTEC FX system across the four bottle types (Plus Aerobic/F, Lytic Anaerobic/F, Mycosis IC/F, Plus Anaerobic/F)

Microorganism species	mTTD in CorneaMax medium (h)			mTTD in Tissue-C medium (h)		
	10 mL	1 mL	*p*-value *	10 mL	1 mL	*p*-value *
**Strict aerobic bacteria**	**80.7**	**26.8**	**ns**	**31.7**	**29**	**ns**
*K. rhizophila*	143.7	39	ns	249	42.6	ns
*P. aeruginosa*	17.7	14.7	ns	20.7	15.3	ns
**Facultative aerobic-anaerobic bacteria**	**173.2**	**113.6**	**ns**	**285.6**	**229**	**0.03**
*S. aureus*	123.8	94.2	ns	215.7	150.9	ns
*S. pyogenes*	122.4	122	ns	336	336	ns
*B. subtilis*	273.8	125.1	0.036	305.1	200	ns
**Strict anaerobic bacteria**	**171.2**	**139.3**	**ns**	**161.7**	**146.6**	**ns**
*B. fragilis*	18.3	23.8	ns	22.3	24.2	ns
*C. sporogenes*	175.2	71.2	ns	126.7	79.7	ns
*C. acnes*	320	322.8	ns	336	336	ns
**Fungi**	**34.2**	**34.4**	**ns**	**50.8**	**56.6**	**ns**
*C. albicans*	46.2	35.5	ns	81	79.7	ns
*A. brasiliensis*	22.3	35.2	0.035	20.5	33.5	0.035
**All microorganisms**	**137.4**	**97.6**	**ns**	**181.2**	**153.5**	**ns**

*Wilcoxon test; ns = not significant; negative bottles were assigned a detection time of 336 h

In CorneaMax, the mTTD was 137.4 h for 10 mL and 97.6 h for 1 mL, while in Tissue-C, the mTTD was 181.2 h for 10 mL and 153.5 h for 1 mL. Although the 1 mL volume tended to shorten the detection times of antibiotic-susceptible microorganisms in both organ culture media, a significant advantage was observed for facultative aero-anaerobic bacteria in Tissue-C (*p*-value = 0.03). In contrast, for *A. brasiliensis*, reducing the inoculation volume to 1 mL significantly increased the mTTD by 12.9 h (*p*-value = 0.035) and 13 h (*p*-value = 0.035) in CorneaMax and Tissue-C media respectively.

## Discussion

In France in 2023, most eye banks relied on the CorneaMax medium for the preservation of corneal grafts. This organ culture medium has been extensively evaluated for its ability to detect microorganisms [26, 27]. In contrast, no comparative study has yet assessed the microbiological performance of the Tissue-C medium. In the context of eye banking, such validation is essential for its implementation in routine clinical practice. In this study, we compared the performance of Tissue-C and CorneaMax for the detection of ten microorganisms using the BD BACTEC FX blood culture system. We found that Tissue-C medium exhibits increased antibiotic activity, which can reduce the detection sensitivity of highly susceptible microorganisms. To overcome this limitation, we developed an optimized microbiological procedure that enhances the detection of microbial contamination in both CorneaMax and Tissue-C media.

Our laboratory ensures microbiological safety for a French eye bank performing approximately 400 corneal preservations per year. In our routine practice, 10 mL of CorneaMax is tested in three blood culture bottles including Plus Aerobic/F, Mycosis IC/F, and Lytic Anaerobic/F, which are incubated for up to 14 days in the BD BACTEC FX system [17, 26]. In this system, two types of anaerobic bottles are available, and their performance varies significantly depending on the clinical context. For the diagnosis of bacteremia, the BACTEC Lytic Anaerobic/F bottle, which contains saponin to enhance blood cells lysis, has demonstrated its superior performance in the blood matrices [28]. The other anaerobic bottle, Plus Anaerobic/F, contains antibiotic-chelating resin that enhances the performance of blood cultures in patients receiving antimicrobial therapy at the time of blood collection [29, 30]. Given that organ culture media contain high concentration of antibiotics, we specifically tested the Plus Anaerobic/F bottle to improve the detection of strict anaerobes, which are usually difficult to recover in the presence of antibiotics. As excepted, we found that resin-containing blood culture bottles improve the detection anaerobes such as of *C. sporogenes* and *C. acnes*. Our previous works also showed that resin-containing bottles enhance the detection of fungal contaminations, likely due to the chelation of amphotericin B present in the organ culture medium [18]. However, Mycosis IC/F bottle support the growth of a variety of pathogenic fungi [31], which represent an increased cause of severe PKIK [14]. Collectively, these results support the use of resin-containing blood culture bottles to ensure the microbiological safety of organ culture medium.

In our study, we first compared the detection of microorganisms in CorneaMax and Tissue-C using an organ culture medium volume of 10 mL per bottle. Strikingly, sensitivity was reduced with Tissue-C compared to CorneaMax. Our results are consistent with a previous studies using different media. Schroeter and colleagues (20) successfully detected six European Pharmacopoeia reference strains using 10 mL of a homemade organ culture medium with a composition comparable to CorneaMax. By contrast, inoculation of 9 mL of Tissue-C into resin-containing BACTEC bottles yielded overall sensitivities of 66.7% and 88.9% at the Rome and Monza eye banks, respectively [32]. To increase the sensitivity of microbiological testing, neutralizing the effect of antibiotics on the growth strains has been successfully achieved using enzymes, such as penicillinase [33]. However, this requires an extra manipulation step during bottle inoculation and is not effective on the streptomycin present in the organ culture medium. Similarly, some cornea banks use the RESEP system developed by ALCHIMIA, which is a syringe containing a chelating resin to remove antimicrobials [32, 34–36]. However, the use of RESEP system adds consumable costs and requires additional hand on time, including 20-minute contact with the RESEP syringe. It also a potential source of contamination during inoculation procedure, which may lead to corneal loss. An alternative approach consists of reducing the antibiotic burden by decreasing the volume of organ culture medium inoculated per bottle, thereby preventing saturation of the chelating resins and allowing effective bacterial growth. Since the European Pharmacopoeia 10.5 recommends an inoculum corresponding to 1% of the total volume for cellular products, we evaluated a 1 mL inoculation volume, as corneas are preserved in 100 mL of organ culture medium. Although reducing the inoculum to 1 mL could theoretically compromise sensitivity, BD BACTEC systems have very low detection limits [37]. In our study, 100% sensitivity was achieved with CorneaMax using a 1 mL inoculum. With Tissue-C, reducing the inoculation volume similarly resulted in 100% sensitivity for eight strains, including *B. subtilis* and *K. rhizophila*, which were not consistently detected with 10 mL. However, even with a 1 mL inoculum, *S. pyogenes* and *C. anceps* were not detected in Tissue-C. In line with the findings of Vignola et al. [32]—who did not test *C. acnes* and *S. pyogenes*—our approach of reducing the volume to 1 mL improved sensitivity similarly to the RESEP system. Nevertheless, in our study, a further reduction of the inoculation volume to 200 µL in a Plus Anaerobic/F bottle was required to ensure the detection of *C. acnes* and *S. pyogenes* in Tissue-C, deviating from the European Pharmacopoeia guidelines.

Interestingly, despite similar compositions and identical antibiotic concentrations, Tissue-C showed higher antimicrobial activity than CorneaMax. Although we did not measure the exact antibiotic levels, this difference may result from variations in antibiotic purity, additional antimicrobials in fetal bovine serum, or interactions with medium components.

Our study highlights that microbiological monitoring of corneal grafts requires careful method optimization tailored to the organ culture medium used in eye banks. Microbiologists should include a broad panel of strains, particularly those highly susceptible to antibiotics. Moreover, the adoption of a new organ culture medium by eye banks requires a comprehensive evaluation, including its ability to preserve cellular integrity [23]. In conclusion, in compliance with European Pharmacopoeia 10.5, we recommend inoculating 1 mL of CorneaMax into three BD BACTEC blood culture bottles (Plus Aerobic/F, Plus Anaerobic/F, and Mycosis-IC/F) for microbiological monitoring. The use of Tissue-C requires an additional Plus Anaerobic/F bottle inoculated with 200 µL.

## Supplementary Information

Below is the link to the electronic supplementary material.

Supplementary Material 1.

## Data Availability

No datasets were generated or analysed during the current study.
