# Supplementary material for: Evaluation of Tissue-C and CorneaMax organ culture media for microbial contamination detection in corneal grafts using the BD BACTEC FX system
Source: Eur J Clin Microbiol Infect Dis. 2026 Apr 27;45(8):2391–9. doi: 10.1007/s10096-026-05459-8 (PMC13428760; doi:10.1007/s10096-026-05459-8)
Supplement: Supplementary file 1 — Supplementary Material 1. [file 10096_2026_5459_MOESM1_ESM.docx]

**Evaluation of Tissue-C and CorneaMax organ culture media for microbial contamination detection in corneal grafts using the BD BACTEC FX system**

Judith Samard ^1^, Martin Fayolle ^1,2,3^, Sandrine Ninotta ^4,5^, Sophie Acquart ^4^, Chloe Talon ^1,2,3^, Manon Lleres-Vadeboin ^1,3,3^, Philippe Gain ^5,6^, Gilles Thuret ^5,6^, Paul O. Verhoeven ^1,2,3#^ and Anne Carricajo ^1,2,3^

^1^ Department of Infectious Agents and Hygiene, Bacteriology-Hygiene Unit, University Hospital of St-Etienne, St-Etienne, France.

^2^ Faculty of Medicine, Jean Monnet University, St-Etienne, France.

^3^ CIRI, Centre International de Recherche en Infectiologie, GIMAP team, INSERM U1111 - CNRS UMR5308 - ENS Lyon - UCBL1, Lyon, France.

^4^ Eye Bank, Auvergne Rhône Alpes French Blood Center, Saint-Etienne, France.

^5^ Laboratory Biology, Engineering and Imaging for Ophthalmology, Faculty of Medicine, University Jean Monnet, Saint-Etienne, France.

^6^ Ophthalmology Department, University Hospital, Saint-Etienne, France.

^#^ Corresponding author: Prof. Paul Verhoeven. Email : paul.verhoeven@univ-st-etienne.fr ; Address: Service des Agents Infectieux et d’Hygiène, CHU de St-Etienne, 42270 St Priest-en-Jarez, France. Phone/Fax number: +33 477829228/+33 477828460.

# Supplementary information

**Supplementary data**

**Supplementary tables**

Table S1

Table S2

# Supplementary data

## Comparison of organ culture medium composition

Briefly, the CorneaMax medium [1] contains vitamins, electrolytes, essential and non-essential amino acids, 2% irradiated fetal calf serum, penicillin (100 IU/mL), streptomycin (0.1 mg/mL), HEPES buffer, bicarbonates, and a phenol red pH indicator. The Tissue-c medium [2] is composed of sodium pyruvate, glucose, amino acids, mineral salts, 2% newborn calf serum, penicillin (100 IU/mL), streptomycin (0.1 mg/mL), amphotericin B (0.25 µg/mL), HEPES buffer, bicarbonates, a phenol red pH indicator, and purified water. Finally, the antibiotic concentrations are then identical. The only difference is the addition of amphotericin B (0.25 µg/mL) in the Tissue-C medium to prevent fungal contamination. However, we noted a slight difference in pH (7.25 ± 0,25 for CorneaMax vs 7.4 ± 0.20 for Tissue-C media) and osmolarity (300 ± 45mOsm/kg for CorneaMax vs 320 ± 20 mOsm/kg for Tissue-C).

[1] Available at https://eurobio-scientific.eu/fr/386-gamme-cornea

[2] Available at https://www.alchimiasrl.com/en/tissue-c/

# Supplementary tables

**Table S1.** Bacterial load of stock inoculum used for the different experiments of the study.

| **Bacterial strain** | **Bacterial load in CFU/ml** | | | |
| --- | --- | --- | --- | --- |
|  | **Replicate 1** | **Replicate 2** | **Replicate 3** | **Mediane [IQR]** |
| **Experiments with a 10 mL inoculation volume** (see Table 1) | | | | |
| *K. rhizophila* ATCC 9341 | 1800 | 600 | 1300 | 1300 [950-1550] |
| *P. aeruginosa* ATCC 9027 | 5000 | 5000 | 1500 | 5000 [3250-5000] |
| *S. aureus* ATCC 6538 | 4400 | 6400 | 1000 | 4400 [2700-5400] |
| *S. pyogenes* CTCB 1031 | 800 | 700 | 1000 | 800 [750-900] |
| *B. subtilis* CIP 5262 | 1500 | 380 | 6000 | 1500 [940-3750] |
| *B. fragilis* ATCC 25285 | 1600 | 530 | 8400 | 1600 [1065-5000] |
| *C. sporogenes* CIP 7939 | 920 | 800 | 500 | 800 [650-860] |
| *C. acnes* clinical strain | 10700 | 5600 | 9200 | 9200 [7400-9950] |
| *C. albicans* ATCC 10237 | 300 | 400 | 1000 | 400 [350-700] |
| *A. brasiliensis* ATCC 1727 | 8200 | 2000 | 3000 | 3000 [2500-5600] |
| **Experiments with a 1 mL inoculation volume** (see Table 2) | | | | |
| *K. rhizophila* ATCC 9341 | 600 | 100 | 1000 | 600 [350-800] |
| *P. aeruginosa* ATCC 9027 | 700 | 800 | 200 | 700 [450-750] |
| *S. aureus* ATCC 6538 | 1000 | 800 | 600 | 800 [700-900] |
| *S. pyogenes* CTCB 1031 | 100 | 300 | 300 | 300 [200-300] |
| *B. subtilis* CIP 5262 | 380 | 100 | 100 | 100 [100-240] |
| *B. fragilis* ATCC 25285 | 200 | 200 | 200 | 200 [200-200] |
| *C. sporogenes* CIP 7939 | 920 | 800 | 500 | 800 [650-860] |
| *C. acnes* clinical strain | 1000 | 1000 | 1000 | 1000 [1000-1000] |
| *C. albicans* ATCC 10237 | 300 | 400 | 1000 | 400 [350-700] |
| *A. brasiliensis* ATCC 1727 | 350 | 350 | 350 | 350 [350-350] |
| **Experiments with a 200 µL inoculation volume** (see result section) | | | | |
| *S. pyogenes* CTCB 1031 | 860 | 40 | 100 | 100 [70-480] |
| *C. acnes* clinical strain | 900 | 900 | 900 | 900 [900-900] |

**Table S2.** Minimum Inhibitory Concentration (MIC) of microorganisms.

| **Bacterial strain** | **MIC (mg/L)** | |
| --- | --- | --- |
|  | **Penicillin G** | **Streptomycin** |
| *S. pyogenes* CTCB 1031 | > 0.016 | 24 |
| *C. acnes* clinical strain | 0.19 | 48 |
| *S. aureus* ATCC 6538 | > 0.016 | 6 |
| *E. faecium* clinical strain | 32 | > 1024 |
